# Antimicrobial Peptides for Therapeutic Applications: A Review

**DOI:** 10.3390/molecules171012276

**Published:** 2012-10-18

**Authors:** Min-Duk Seo, Hyung-Sik Won, Ji-Hun Kim, Tsogbadrakh Mishig-Ochir, Bong-Jin Lee

**Affiliations:** 1 College of Pharmacy, Ajou University, Suwon 443-749, Korea; 2 Department of Biotechnology, College of Biomedical and Health Science, Konkuk University, Chungju, Chungbuk 380-701, Korea; 3 Center for Structural Biology and Departments of Biochemistry and Pharmacology, Vanderbilt University School of Medicine, Nashville, TN 37232, USA; 4 Faculty of Biology, National University of Mongolia, Ulaanbaatar Box 377, Mongolia; 5 Research Institute of Pharmaceutical Sciences, College of Pharmacy, Seoul National University, Seoul 151-742, Korea

**Keywords:** antimicrobial peptides (AMPs), antibiotics, structure-activity relationship (SAR)

## Abstract

Antimicrobial peptides (AMPs) have been considered as potential therapeutic sources of future antibiotics because of their broad-spectrum activities and different mechanisms of action compared to conventional antibiotics. Although AMPs possess considerable benefits as new generation antibiotics, their clinical and commercial development still have some limitations, such as potential toxicity, susceptibility to proteases, and high cost of peptide production. In order to overcome those obstacles, extensive efforts have been carried out. For instance, unusual amino acids or peptido-mimetics are introduced to avoid the proteolytic degradation and the design of short peptides retaining antimicrobial activities is proposed as a solution for the cost issue. In this review, we focus on small peptides, especially those with less than twelve amino acids, and provide an overview of the relationships between their three-dimensional structures and antimicrobial activities. The efforts to develop highly active AMPs with shorter sequences are also described.

## 1. Introduction

Antimicrobial peptides (AMPs) are endogenous polypeptides produced by multicellular organisms in order to protect a host from pathogenic microbes. AMPs are also defined as host defense peptides because of their essential role in constituting the innate immunity system [1,2,3,4]. AMPs are generally comprised of less than 50 amino acids approximately, and characterized by cationic amphipathic properties. In general, when AMPs are folded in membrane mimetic environments, one side of AMPs is positively charged (mainly due to lysine and arginine residues) and the other side contains a considerable proportion of hydrophobic residues [1,2,5,6]. 

AMPs show broad-spectrum antimicrobial activities against various microorganisms, including Gram-positive and Gram-negative bacteria, fungi, and viruses [1]. Of particular interest, many AMPs are effective against multi-drug resistant (MDR) bacteria and possess low propensity for developing resistance [7,8,9]. Bacterial resistance to antibiotics can be achieved by diverse routes including inhibition of the drug-target interaction, modification of the drug-binding site in target proteins, and efflux of the drug from target cells [10]. Microorganisms can also alter their genetic patterns in response to environmental changes using their own complex systems called sensor-transducer response systems. For instance, bacteria can modify their gene expression in the presence of AMPs [11]. AMPs possess low propensity for developing resistance, probably due to their distinguished mode of action. Most AMPs, with their amphipathic nature, directly act on the membrane of the pathogen. The cationic properties of AMPs are implicated in their selective interaction with the negatively charged surfaces of microbial membranes, resulting in the accumulation of AMPs on the membrane surface. Then, their hydrophobic portions are responsible for the interaction with hydrophobic components of the membrane. From this complex interaction with the membrane, major rearrangements of its structure occur, which may result from the formation of peptide-lipid specific interactions, the peptide translocation across the membrane and interaction with intracellular targets or the most common mechanism, a membranolytic effect [3,11,12,13,14,15,16]. Such a characteristic mechanism of action, distinct from that of conventional antibiotics, enables AMPs to avoid the common resistance mechanisms observed for classic antibiotics. Consequently, AMPs are receiving great attention as promising alternatives to conventional antibiotics to overcome the current drug resistance crisis. In this review, we describe the advantages and limitations of AMPs as novel antibiotic agents and structural information aids for developing new AMPs. Especially, we focus on small peptides (less than twelve amino acids in length) in clinical trials and their structure-activity relationships (SAR). 

## 2. Structure-Activity Relationships (SAR) of Antimicrobial Peptides

AMPs can be classified into four groups based on their structures: α-helical peptides, β-sheet peptides, extended peptides, and loop peptides [1,2,3,6]. The α-helical AMPs, including magainin, cecropin, and pexiganan, constitute a representative class of AMPs that are the most well established in structure-activity relationships. This group of peptides is usually unstructured in aqueous solution and forms amphipathic helices in membranes or membrane-mimicking environments. Most α-helical AMPs disrupt bacterial membranes, and several mechanisms of action employed by various AMPs have been proposed. The α-helical amphipathic peptides form barrel-like bundles in the bacterial membranes, and these transmembrane clusters line amphipathic pores (barrel-stave model). Many α-helical AMPs, including some magainins and cecropins, can dirupt bacterial membranes by forming carpet-like clusters of peptides. The peptides adsorb and align in parallel to the surface of bacterial membranes, then the membranes are collapsed into micelle-like structures by high concentrations of peptides (carpet model). The α-helical AMPs, such as some magainins and protegrins, form toroidal pores to disrupt the bacterial membranes (toroidal pore model) [1,2,3,4,5,6,7,9,11].

The β-sheet AMPs, such as α-, β-defensins, and protegrin, are stabilized by disulfide bridges, and form relatively rigid structures. Many of β-sheet AMPs exert their antimicrobial activities by disrupting bacterial membranes. They are perpendicularly inserted or tilted into the lipid bilayer to form toroidal pores, and hydrophilic regions of the peptides are associated with the polar head groups of the membranes [3]. 

The extended AMPs, which are predominantly rich in specific amino acids such as proline, tryptophan, arginine, and histidine, have no regular secondary structure elements. Indolicidin is a tryptophan/proline-rich extended peptide and Bac5 and Bac7 are proline/arginine-rich peptides [17,18]. Many extended AMPs are not active against the membranes of pathogens, but they can achieve their antimicrobial activities by penetrating across the membranes and interacting with bacterial proteins inside [3]. On the other hand, some extended peptides, such as indolicidin, are membrane active and induce membrane leakage. Indolicidin is a 13-amino acid AMP containing five tryptophan and three proline residues. The peptide adopts a poly-L-II helical structure in the presence of liposomes, and the high content of tryptophan residues is responsible for the interaction with lipid membranes [17]. The loop AMPs, including bactenecin, adopt a loop formation with one disulfide bridge.

Understanding the structure-activity relationships (SAR) of AMPs is essential for the design and development of novel antimicrobial agents with improved properties. In particular, the atomic level structures of AMPs can provide versatile information for all stages of drug development, including the peptide design and modification for pharmaceutical application. Pexiganan (also known as MSI-78), a synthetic variant of magainin 2, is one of the best-investigated AMPs in terms of drug development [19,20]. Pexiganan has reached clinical trials as a novel topical broad-spectrum antibiotic for the treatment of mild-to-moderate diabetic foot ulcer infections [4,20,21]. The three-dimensional structure of pexiganan, determined by Nuclear Magnetic Resonance (NMR) spectroscopy, revealed that the peptide forms a dimeric antiparallel α-helical structure in the presence of membrane mimetics [22,23]. The atomic resolution structure also demonstrated that the side chains of three phenylalanine residues are important for the self-dimerization [23]. In addition to the peptide structure, its orientation in membrane is critical to understand the exact mode of membrane interaction. A solid-state NMR study of pexiganan suggested that the peptide adopts a helical conformation interacting with the membrane surface and then the dimeric peptide is inserted into the membrane [22].

Knowledge on the functional structures of AMPs also enables a rational design of synthetic model peptides. In this respect, minimalistic *de novo* approaches to design model amphipathic helical peptides are noteworthy. For example, certain 14- or 15-mer peptides (namely LK peptides) composed of two kinds of amino acids (leucines and lysines), have been characterized as possessing strong antimicrobial activity [24,25]. Then, by introducing a single tryptophan residue at a specific position, 9- to 11-mer LKW peptides could exhibit antimicrobial activity. Now, the *de novo* designed model peptides remain to be further improved by optimizing the amino acid sequence to enhance stability and to reduce potential toxicity. In addition, their activities need to be checked using clinically isolated bacteria. In the LKW peptide design, the tryptophan residue was specifically incorporated in expectation of structural role for conferring activity [26]. Tryptophan is known to stabilize the helical conformation and membrane interaction of peptides. The specific position where the tryptophan should be incorporated could be determined in the helical wheel projection, by aids of structural information [27,28,29,30]. Three-dimensional structures of engineered peptides could provide useful information for the design of shorter and more potent AMPs. Taken together, all these structural studies contribute to a better understanding of the mechanism of action employed by AMPs, and fine structures of natural and synthetic peptides can be used as a scaffold to generate novel AMPs more suitable for pharmaceutical applications. 

## 3. Antimicrobial Peptides as Potential Therapeutics

AMPs are fascinating targets as novel antibiotics because of their broad-spectrum activity, which include drug-resistant bacteria. Since the isolation of magainins from frog skin in 1987 [31], there have been many attempts to develop antibiotics from the natural AMPs. However, despite the efforts over more than two decades, there is no AMP agent currently approved by Food and Drug Administration (FDA) [4,32,33]. Although AMPs have considerable advantages for therapeutic applications, including broad-spectrum activity, rapid onset of activity, and relatively low possibility of resistance emergence, they also have some limitations for drug development. The natural AMPs are labile, depending on the surrounding environments, such as the presence of protease, pH change, and so on [34,35,36]. Other obstacles for the use of peptide antibiotics are the potential toxicity of AMPs for oral application and high cost of peptide production [2]. In general, many AMPs are considered to be less toxic to eukaryotes, but systematical toxicity of AMPs for oral application has not been evaluated. In order to overcome those obstacles, many methods have been proposed. For instances, introduction of unusual amino acids (mainly D-form amino acids) or modification of the terminal regions (acetylation or amidation) improved the stability of peptides by preserving them from proteolytic degradation [36,37]. Also, the use of efficient drug delivery systems, such as liposome encapsulation, can be effective for the improvement of the stability and reduction of potential toxicity [38,39]. Presumably, the practical obstacle may be the cost issue. Production costs are estimated to be roughly $50-$400 per 1 gram of amino acid when running commercial quantities [7]. The most certain solution for the high production cost would be the reduction of the peptide size with retention of the activity. Actually, there have been several successful examples of peptide engineering to reduce the peptide size with improved antimicrobial activity. As an example, the inactive, eleven-residue fragment of gaegurin 5 (GGN5^N11^) could recover the antimicrobial activity by a single tryptophanyl substitution at the hydrophobic-hydrophilic interface of the amphipathic helix [29,30]. 

## 4. Small Peptides in Drug Development

### 4.1. hLF1-11 (Human Lactoferrin 1-11)

Lactoferrin (LF) is an iron-binding glycoprotein which is responsible for part of the innate defense system. The LF can not only bind and trap Fe^3+^ ion, but also interact with the bacterial membrane directly, which enables the LF to possess antibacterial activity [40,41,42,43]. The synthetic hLF1-11 peptide (GRRRRSVQWCA) is a lactoferrin derivative corresponding to the N-terminal eleven residues of human lactoferrin. The hLF1-11 peptide shows antimicrobial activity against both Gram-positive and -negative bacteria and various fungi. The synthetic peptide is also effective against methicillin-resistant *Staphylococcus aureus* (MRSA) and multidrug-resistant *Acinetobacter baumannii* strains [43,44,45]. Moreover, hLF1-11 is active against fluconazole-resistant *Candida albicans*, and can be used with classic antibiotics for a synergistic effect. Preincubation of fluconazole-resistant *C. albicans* with hLF1-11 significantly enhances the candidacidal effect of fluconazole [46].

The three-dimensional structures of several lactoferrin-derived peptides in membrane mimetic conditions have been solved by NMR spectroscopy [47,48,49,50]. The solution structure of LF11 (FQWQRNIRKVR), another N-terminal fragment based on human lactoferrin, showed conformational differences between in an anionic detergent (sodium dodecylsulfate; SDS) and in a zwitterionic detergent (dodecylphosphocholine; DPC) micelles. The structure of LF11 in SDS micelles was well defined and the tryptophan residue was significantly protected in the micelles. However, in DPC micelles, the structure was less defined and the tryptophan residue was less protected [48]. The conformation of hLF1-11 (GRRRRSVQWCA) also varied depending on the environments [43]. Molecular Dynamics (MD) simulation results of hLF1-11 in various solvents suggested that the peptide should be categorized as a loop peptide and adopts a more favorable conformation for the membrane interaction in membrane-mimicking environments. In particular, cationic residues (‑RRRR‑) of the hLF1-11 were rather flexible to be suitable for the interaction with the anionic bacterial membrane. Furthermore, the hydrophobic region, which was positioned approximately perpendicular to the cationic residues, enabled the peptide to bind to the membrane interior. Taken together, all these studies elucidated the structural selectivity of AMPs between bacterial and eukaryotic membranes.

The safety and tolerability of hLF1-11 in healthy volunteers and haematopoietic stem cell transplantation (HSCT) recipients have been tested [43,51]. Intravenous administrations of hLF1-11 were safe and well tolerated in both healthy volunteers and the HSCT recipients. Although some adverse events (AE) were reported, all of them were mild in intensity and reversible. Pharmacodynamic evaluations including cytokine measurements also showed no significant changes in both populations. 

### 4.2. (CKPV)_2_ Peptide (α-MSH Derivative, also Named CZEN-002)

The α-melanocyte stimulating hormone (α-MSH; SYSMEHFRWGKPV), obtained from the cleavage of pro-opiomelanocortin (POMC), is a neuropeptide hormone showing anti-inflammatory activity and antimicrobial activity [52,53,54]. It is noteworthy that α-MSH peptides are effective against *Candida albicans* and distinct in action mechanism from other natural AMPs. The candidacidal activity of the α-MSH peptides is caused by increased cyclic adenosine monophosphate (cAMP) in the *C. albicans* cells [55], whereas most AMPs kill the bacteria through direct interaction with the bacterial membrane. It has been known that cAMP-mediated modulation is essential for gene expression in *C. albicans* and the cAMP-activating effect of α-MSH interferes with the cAMP-mediated signaling pathway [56,57]. 

Interestingly, the C-terminal tripeptide (α-MSH_11-13_; KPV) also has anti-inflammatory and antimicrobial activities similar to those of the full-length α-MSH [55,58]. The synthetic peptide (CKPV)_2_, also named CZEN-002, was designed based on the KPV (α-MSH_11-13_) peptide. The peptide is a dimeric octamer peptide comprising two units of KPV peptide connected by a cysteine-cysteine linker, which is classified as loop peptide. The (CKPV)_2_ peptide revealed outstanding candidacidal activity against *C. krusei* and *C. glabrata* that are emerging as drug-resistant strains, but it showed very low toxicity to host cells [59,60,61]. (CKPV)_2_ peptide also exerts anti-inflammatory effects as well as the candidacidal activity. It can inhibit TNF-α production with comparable activity to that of the potent α-MSH analogues [60,62].

Because of the simple sequence and small size as well as the excellent candidacidal activity, (CKPV)_2_ is regarded as a promising agent for development of candidacidal and anti-inflammatory drugs. (CKPV)_2_ is being currently evaluated in clinical trials for treatment of vulvovaginal candidiasis [4]. In addition, the structure of (CKPV)_2_ can be a valuable target for the design of other short antimicrobial peptides. Thus, the three-dimensional structure of the (CKPV)_2_ was determined by NMR spectroscopy, to identify a novel scaffold for the design of small compounds having candidacidal activity [59]. (CKPV)_2_ adopts a symmetric dimer with an extended backbone structure, which resembles the model structure of natural α-MSH peptide [63]. The overall conformation of (CKPV)_2_ showed a β-turn-like fold, which may be critically related to the higher activity of (CKPV)_2_ than KPV monomer [59].

### 4.3. P-113 (Histatin 5 Derivative, Also Named PAC113)

Histatin 5, a parent molecule of P-113, is a small cationic peptide secreted into the saliva. Among the histatin family peptides, the histatin 5 exerts the most potent antimicrobial activity against bacteria and fungi [64,65]. The P-113 is an optimized fragment of the histatin 5 comprising twelve residues (residues 4-15 of histatin 5; AKRHHGYKRKFH), and retains antibacterial and anticandidal activity similar to that of the parent molecule, histatin 5 [66]. P-113 has potent candidacidal activity against various strains of *C. albicans*, *C. glabrate*, *C. parapsilosis*, and *C. tropocalis* that cause oral candidiasis. In addition to the broad spectrum of anticandidal activity, P-113 is also active against fluconazole resistant *C. albicans* and *C. glabrata* [66], which suggests that P-113 can be a promising antifungal agent for the treatment of oral candidiasis. 

Structural investigations of hsitatin 5 and P-113 in various solvents have been performed mainly by NMR spectroscopy [67,68,69,70]. The three-dimensional solution structures of histatin 5 revealed that the peptide prefers α-helical conformation in DMSO (dimethyl sulfoxide) or TFE (trifluoroethanol)/water, while it remains unstructured in water [68,69]. However, the helical conformation of histatin 5 in TFE/water system consists of two α-helices, one from Ser2 to Gly9 and the other from Lys11 to His19, whereas a single-alpha helix is formed in DMSO. MD simulation results of histatin 5 also supported that the α-helical regions of the peptide remain structured in the TFE system, but gradually unfold in water [70]. All the data suggest that the adaptation and maintenance of helical structure is essential for the antimicrobial activity of histatin 5.

Structural properties of P-113, which are related to the antimicrobial activity, are also similar to those of histatin 5. Structural conversion of the P-113 from a disordered conformation in aqueous solution to an α-helical conformation in membrane mimetic environments was evidenced by a circular dichroism study [66,67]. The relationships between α-helical propensity and antimicrobial activity of P-113 can be demonstrated by comparing the structures and activities between the free- and metal bound-P-113 peptides. The solution structure of Zn(II)-bound P-113 (Zn(II)-P-113) contains a well defined *N*-terminal region (from Ala1 to His5) and a less defined C-terminal region (from Gly6 to His12). Imidazole rings of all three His residues (His4, His5, and His12) are coordinating one Zn(II) ion. The involvement of His12 in metal coordination makes the peptide to have lower propensity to adopt an alpha-helical conformation in C-terminal region, therefore resulting in the decrease of antimicrobial activity. The antimicrobial activity of Zn(II)-P-113 against a variety of microorganisms, including *S. aureus*, *E. faecalis*, and *C. albicans*, is much lower than that of metal free P-113 [71]. 

## 5. Conclusions

The serious problems caused by drug resistant bacteria have created an urgent need for the development of alternative therapeutics. In this respect, AMPs are considered as promising antimicrobial agents for producing new generation antibiotics. Additionally, atomic level structures of AMPs are prerequisite information for the generation of improved peptide antibiotic candidates. Although there are several obstacles to be overcome for clinical applications, natural and synthetic AMPs are still attractive sources to the pharmaceutical companies. In order to facilitate commercial development of peptide antibiotics, it is reasonable to focus on small peptides. Successful generation of short antimicrobial peptide molecules includes the A4W-GGN5^N11^ [29,30], hLF1-11 [43,44,45,46,47,48,49,50,51], (CKPV)_2_ [58,59,60,61,62], P-113 [66,67,71], and LKW [24,25,26,27] peptides. In terms of production cost, the use of those peptides would be advantageous in the pharmaceutical field. 
